# Regulation of Human Formyl Peptide Receptor 1 Synthesis: Role of Single Nucleotide Polymorphisms, Transcription Factors, and Inflammatory Mediators

**DOI:** 10.1371/journal.pone.0028712

**Published:** 2011-12-09

**Authors:** Heini M. Miettinen

**Affiliations:** Department of Microbiology, Montana State University, Bozeman, Montana, United States of America; Louisiana State University, United States of America

## Abstract

The gene encoding the human formyl peptide receptor 1 (*FPR1*) is heterogeneous, containing numerous single nucleotide polymorphisms (SNPs). Here, we examine the effect of these SNPs on gene transcription and protein translation. We also identify gene promoter sequences and putative *FPR1* transcription factors. To test the effect of codon bias and codon pair bias on FPR1 expression, four *FPR1* genetic variants were expressed in human myeloid U937 cells fused to a reporter gene encoding firefly luciferase. No significant differences in luciferase activity were detected, suggesting that the translational regulation and protein stability of FPR1 are modulated by factors other than the SNP codon bias and the variant amino acid properties. Deletion and mutagenesis analysis of the *FPR1* promoter showed that a CCAAT box is not required for gene transcription. A −88/41 promoter construct resulted in the strongest transcriptional activity, whereas a −72/41 construct showed large reduction in activity. The region between −88 and −72 contains a consensus binding site for the transcription factor PU.1. Mutagenesis of this site caused significant reduction in reporter gene expression. The PU.1 binding was confirmed *in vivo* by chromatin immunoprecipitation, and the binding to nucleotides −84 to −76 (TTCCTATTT) was confirmed *in vitro* by an electrophoretic mobility shift assay. Thus, similar to many other myeloid genes, *FPR1* promoter activity requires PU.1. Two single nucleotide polymorphisms at −56 and −54 did not significantly affect *FPR1* gene expression, despite differences in binding of transcription factor IRF1 *in vitro*. Inflammatory mediators such as interferon-γ, tumor necrosis factor-α, and lipopolysaccharide did not increase *FPR1* promoter activity in myeloid cells, whereas differentiation induced by DMSO and retinoic acid enhanced the activity. This implies that the expression of FPR1 in myeloid cells is developmentally regulated, and that the differentiated cells are equipped for immediate response to microbial infections.

## Introduction

Formyl peptide receptor 1 (FPR1) is a G protein-coupled receptor that mediates important host defense functions such as chemotaxis and killing of microorganisms through phagocytosis and oxidative burst [1]. The coding sequence of *FPR1* contains ten single nucleotide polymorphisms (SNPs); six are non-synonymous, resulting in amino acid changes, and four are synonymous [2]–[4]. Most of the SNPs do not exhibit strong linkage disequilibrium, resulting in a large number of variants, with >30 sequenced haplotypes identified in Caucasians so far [4]. GenBank reports an additional 7 SNPs (http://www.ncbi.nlm.nih.gov/SNP/snp_ref.cgi?locusId=2357), but most of them have not yet been validated. FPR1, which contains 350 amino acids, could theoretically be encoded in >10^183^ ways, with each adjacent pair of amino acids encoded by 2–36 different pairs of synonymous codons. However, some codons are used more or less frequently, indicating a certain codon bias [5]. For example, in humans, GTG is used 4 times more frequently than GTA to encode valine, and CTG is used 5.1 times more frequently than TTA to encode leucine (http://www.kazusa.or.jp/codon/cgi-bin/showcodon.cgi?species=9606). Similarly, codon pairs are used more or less frequently than expected, but not always following the codon bias frequencies. Based on the codon frequencies mentioned above, the amino acid pair Val-Leu is expected to be encoded by GTG-CTG much more frequently than GTA-TTA, but in fact this sequence is encoded somewhat less frequently by GTG-CTG than by GTA-TTA (codon pair bias scores of 0.144 and 0.397, respectively) (www.sciencemag.org/cgi/content/full/320/5884/1784/DC1; [6]). A study of the poliovirus capsid protein showed compelling evidence that codon pair usage affects protein translation: Large DNA molecules containing over- or underrepresented synonymous codon pairs encoding poliovirus capsid protein were expressed in human HeLa cells and the rate of protein translation was measured; DNA with underrepresented codon pairs caused decreased rates of protein translation and attenuation of poliovirus [6]. The reason for the poor translation efficiency is thought to be certain tRNAs that interact poorly on the ribosomal A- and P-sites of underrepresented codon pairs [7]. Similarly, the poor translation efficiency in the presence of infrequent codons is thought to be the limiting amount of tRNAs [8]. Since we have previously observed variable expression levels of FPR1 in neutrophils from human donors, we investigated the possibility that certain combinations of *FPR1* SNPs may affect the quantity of translated FPR1.

In addition to translation efficiency, protein expression levels depend on other factors such as gene transcription, mRNA stability, and protein stability. Relatively little is currently known about the role of these factors on the regulation of FPR1. A study using thioglycolate-elicited mouse peritoneal macrophages and neutrophils showed increased *FPR1* mRNA stability upon exposure to lipopolysaccharide (LPS) and a barely detectable increase in *FPR1* gene transcription [9]. To further explore the control of FPR1 expression at the level of gene transcription, we determined the minimal functional *FPR1* promoter, studied the role of two SNPs on transcriptional regulation, and examined the binding of putative transcription factors to the core promoter. We also confirmed that differentiation of human myeloid U937 cells with DMSO and retinoic acid increases FPR1 expression [10], [11]. However, unlike many cell surface proteins involved in innate immune defense, FPR1 expression does not appear to be transcriptionally induced in response to activators such as tumor necrosis factor-α (TNFα), lipopolysaccharide (LPS) and interferon-γ (IFNγ), suggesting that *FPR1* transcription is controlled by cell differentiation rather than inflammatory activators. This concurs with the observed distribution of FPR1 in band cells, segmented cells and polymorphonuclear neutrophils (PMNs) [12].

## Materials and Methods

### Human subjects

A total of 69 Caucasians from the Montana State University Blood Donor Program participated in the study. The study was approved by the Institutional Review Board of Montana State University and the blood donors gave their informed written consent to the study.

### Construction of pGL4.10[luc2] reporter plasmids

Genomic DNA was isolated from healthy donors from 250 µl whole blood using E.Z.N.A. Blood DNA Kit II according to the manufacturer's instructions (Omega Biotek). FPR1 haplotypes 8A, 11A, 12D and 16A were amplified by PCR, cloned into pGEM®-T Easy vector (Promega) and sequenced [4]. The *FPR1* inserts were excised with *Eco* RI, subcloned into pBGSA vector, and the correct orientations of the inserts were verified by restriction mapping. The pBGSA mammalian expression vector (GenBank Accession #AY6607190) contains a hybrid SRα promoter composed of the simian virus 40 early promoter and the R-U5 segment of human T-cell leukemia virus type 1 long terminal repeat [13]. The SRα promoter was used to drive expression of the FPR1-firefly luciferase fusion protein. The promoter and the full-length *FPR1* cDNA were amplified by PCR using primers containing restriction sites *Kpn* I and *Bgl* II to allow subcloning in frame with the *luc2* gene in the pGL4.10[*luc2*] vector (Promega; GenBank Accession #AY738222).

### Human FPR1 promoter amplification and sequencing

The promoter sequence of *FPR1* was amplified from 100 ng genomic DNA by PCR. Amplification utilized the following primer pair: “FPR prom −630F25” and “FPR prom +117R23”, where the first nucleotide of the primer is indicated by its position relative to the guanidine (+1) in the transcriptional start site [14], followed by F or R for forward or reverse and the number of nucleotides in the primer. SNP genotypes were identified by direct sequencing of the PCR product; haplotypes were verified after ligation of the PCR amplicons into pGEM®-T Easy.

### Luciferase vector construction

Reporter vectors were constructed in the pGL3 Basic luciferase vector (Promega; GenBank Accession # U47295). The desired promoter regions (−395/41, −274/41, −149/41, −140/41, −122/41, −105/41, −88/41, −72/41 and −50/41) were amplified by PCR using the −56C/−54G haplotype as template. The forward primers included an *Xho* I-site and the reverse primer included a *Hind* III-site for subcloning into pGL3 Basic. The *FPR1* promoter −88/41 −56T/−54C was constructed as above using a −56T/−54C variant as template. *FPR1* promoter constructs −88/41 −56C/−54C and −88/41 −56T/−54C were created by QuickChange™ site-directed mutagenesis using pGL3 Basic-*FPR1* promoter −88/41 −56C/−54G as template (Stratagene). To remove putative transcription factor binding sites, mutations in pGL3 Basic-*FPR1* −149/41 (−56C/−54G) were created using mutagenic primers as follows: NF-Y, 5′(-140) -GCAGACAGTATATTAATGTATTCTTGGGG-3′; PU.1, 5′(-95) -GAAGCTCAGACTTAATATTTCCTGCTACC-3′; STAT-4, 5′(-91) -CTCAGACTTCCTATGGCCTGCTACCCAG-3′. Mutated sequences are underlined. All constructs were confirmed by sequencing.

### Transient transfection and dual luciferase assay of U937 cells

U937 cells (ATCC® Number: CRL-1593.2™) were resuspended at a density of 1.5×10^6^ cells/ml in RPMI-1640 supplemented with 10% FBS, 50 U/ml penicillin and 50 µg/ml streptomycin. 400 µl cell suspension was added to electroporation cuvettes (0.4 cm gap), followed by 20 µg luciferase reporter plasmids (or as indicated in the figure legends) and 300 ng pRL-TK vector (to normalize transfection efficiency) (Promega; Accession # AF025848). Cells were electroporated using a BTX ECM®399 pulse Generator with Personal Electroporation Pak 1 at 200 V, 1050 µF, and moved to wells in a 24 well plate containing 400 µl RPMI-1640 supplemented with 10% FBS, 50 U/ml penicillin, and 50 µg/ml streptomycin. Cells were grown as indicated in the figure legends with or without DMSO and activating factors. Transfected cells were assayed after 24–48 h (as indicated in figure legends) for firefly and *Renilla* luciferase activity using the Dual-Luciferase Reporter Assay System (Promega) in a Berthold EG&G Lumat Luminometer LB 96V [15].

### Chromatin immunoprecipitation (ChIP) and quantitative real-time PCR (q-PCR)

2.5×10^7^ U937 cells were plated at a density of 1.2×10^6^ cells/ml and grown for 24 h before each experiment. Cells were incubated for 10 min at room temperature with 0.1 volume of cross-linking mix (11% formaldehyde, 100 mM NaCl, 0.5 mM Na-EGTA and 50 mM Na-HEPES, pH 8.0), and the reaction was quenched by the addition of 0.125 M glycine (final concentration). Cells were washed with Dulbecco's PBS containing 450 µM CaCl_2_, 245 µM MgCl_2_, 0.1% dextrose and 0.1% BSA. To prevent proteolysis, cells were incubated for 15 min on ice with diisopropyl fluorophosphate (DFP), washed as above and lysed in 1% SDS, 10 mM Na-EDTA (pH 8.0), 50 mM Tris-HCl (pH 8.0), 1 mM PMSF, and a mammalian protease inhibitor cocktail (Sigma). After 5 min incubation on ice, samples were sonicated 14×10 sec on setting 5 with a 50 Sonic Dismembrator (Fisher Scientific) to obtain chromatin with an average size of about 600–800 bp (as judged by gel electrophoresis). The sample was centrifuged 15 min at 20,800× g at 4°C to remove cell debris, and an aliquot of the supernatant was reserved for input in PCR analysis. The rest of the supernatant was diluted with a buffer containing 1% Triton X-100, 150 mM NaCl, 2 mM Na-EDTA (pH 8.0), 20 mM Tris-HCl (pH 8.0), 1 mM PMSF and protease inhibitor cocktail. Samples were incubated on a rotator overnight at 4°C with antibody against PU.1 (sc22805X; Santa Cruz Biotechnology) or with an irrelevant antibody as a negative control. Antibody-DNA complexes were precipitated with Protein A-agarose beads (Sigma) previously blocked with BSA and salmon sperm DNA (to reduce background binding). The beads were washed three times with wash buffer #1 (1% Triton X-100, 0.1% SDS, 150 mM NaCl, 2 mM EDTA, 20 mM Tris-HCl, pH 8.0+protease inhibitors), once with wash buffer #2 (same as #1 with NaCl increased to 500 mM), and finally with wash buffer #3 (20 mM Tris-HCl, pH 8.0, 1 mM EDTA, 250 mM LiCl, 0.5% NP-40, 0.5% Na-deoxycholate, + protease inhibitors). The immune complexes were eluted from the beads by 30 min incubation at 37°C with 100 mM NaHCO_3_, 1% SDS, and the supernatants were treated for 30 min with 500 µg/ml RNase A and 500 µg/ml Proteinase K at 37°C. The cross-links were reversed after addition of 200 mM NaCl at 65°C overnight. DNA was purified by phenol-chloroform-isoamylalcohol (25∶24∶1) extraction and precipitated by ethanol in the presence of linear polyacrylamide carrier. The sonicated input DNA was treated as above, starting with the RNaseA and Proteinase K incubation. Precipitated DNA was resuspended in sterile water. qPCR was carried out from the affinity-precipitated chromatin using Quantace 2×Sensimix and primers corresponding to the promoter region of *FPR1* (nucleotides −87 to 237). The PCR product of 324 bp was quantified using Rotor-Gene software and visualized by 1.5% agarose gel electrophoresis.

### In vitro translation of human PU.1 and IRF1 and electrophoretic mobility shift assay (EMSA)

The human PU.1 and IRF1 cDNAs were amplified by reverse transcriptase PCR from human neutrophil total RNA using forward primers spanning the start sites and including a *Sal* I restriction site, and reverse primers spanning the stop site and including a *Bam* HI restriction site. The amplified PCR products were cloned into pGEM® T Easy and the sequences were confirmed. The cDNAs were subcloned into *Sal* I/*Bam* HI site in pSP64 poly(A) (Promega) and *in vitro* transcribed and translated using the TNT® SP6 high-yield wheat germ protein expression system in the presence of [^35^S]methionine according to manufacturer's protocol (Promega). A negative control reaction was carried out using the pSP64 poly(A) plasmid in the absence of a cDNA insert (^35^S-control). The ^35^S-PU.1 and ^35^S-IRF1 products were electrophoresed on a SDS-polyacrylamide gel, and the gel was subjected to autoradiography to confirm the correct molecular masses of the proteins. Double-stranded oligonucleotide probes spanning *FPR1* promoter region −101 to −63 (containing a putative PU.1 binding site) and −73 to −44 (containing a putative IRF1 binding site) were incubated for 20 min at room temperature in the absence or presence of ^35^S-PU.1, ^35^S-IRF1, or ^35^S-control reaction in a buffer containing 10 mM Tris-HCl, pH 7.6, 50 mM KCl, 1 mM MgCl_2_, 5% glycerol, 0.5 mM EDTA, 1 mM DTT and 100 ng/µl sonicated salmon sperm DNA. Samples were run using the Mini-PROTEAN® 3 cell (Bio-Rad) on 6% non-denaturing polyacrylamide gels (19∶1) in 0.5× TBE at 100 V for 90 min. Gels were fixed and subjected to autoradiography. Control double-stranded oligonucleotide for PU.1 was derived from the promoter region of *gp91^phox^* [16], and control IRF1 double-stranded oligonucleotide was designed based on the published IRF1 binding consensus sequence, flanked by *FPR1* promoter sequence [17].

### Flow cytometry

U937 cells at a concentration of 2.5×10^5^ cells/ml were incubated for 0–5 days in the presence of 1% DMSO. Cells were pelleted by centrifugation and suspended in cold PBS containing 5% FBS, 20 nM *N*-formyl-Nle-Leu-Phe-Nle-Tyr-Lys-fluorescein (a ligand that binds FPR1), and 1 µg/ml propidium iodide (a fluorescent dye used to measure cell viability). Cells were incubated for 1 h on ice, followed by analysis of 10,000 cells using a BD Biosciences FACSCalibur flow cytometer. The scatter plots were gated to show the percentage of cells that did not bind ligand (FPR1 negative cells), cells that bound ligand (FPR1 positive cells) and non-viable cells (propidium iodide positive cells).

## Results

### SNP codon bias and codon pair bias do not affect the expression levels of FPR1

**Figure 1:**
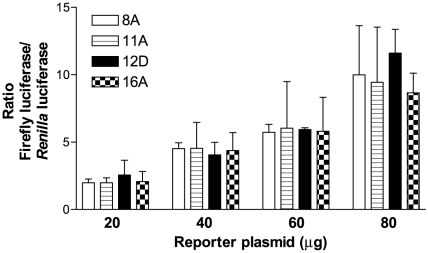
Four FPR1 variants show similar expression levels. FPR1 haplotypes 8A, 11A, 12D and 16A were expressed as fusion proteins with firefly luciferase in U937 cells. Cells were electroporated with various amounts of the firefly luciferase reporter plasmid (as shown) and 300 ng pRL-TK *Renilla* luciferase control reporter plasmid. 24 h post-transfection cell extracts were analyzed using the Promega dual luciferase assay kit. The graphs show the mean ratios of firefly and *Renilla* luciferase from five separate experiments ± S.E.M. One-way analysis of variance showed no statistical differences between the haplotypes.

As mentioned in the Introduction, codon bias and codon pair bias affect the transcription and translation of both prokaryotic and eukaryotic proteins. To examine whether FPR1 haplotypes previously amplified and sequenced by us have variable protein expression, the scores for the various SNP combinations were calculated [4]. As shown in Table 1, which contains a partial list of the 31 haplotypes, the differences based on codon bias were relatively small, whereas the codon pair bias scores showed larger variation. Based on the results, we selected two haplotypes from opposite ends of the scores; haplotypes 8A and 11A as FPR1 variants predicted to have low expression levels, and 12D and 16A as FPR1 variants predicted to have high expression levels on the basis of codon pair bias scores. The coding sequences linked to a strong promoter were inserted into the pGL4.10 [*luc*2] vector to create *FPR1-luciferase* fusions. Human myeloid U937 cells were co-transfected with various amounts of these plasmids and a constant amount of the pRL-TK vector which drives the expression of *Renilla* luciferase under the TK promoter (as an internal standard for transfection normalization). As shown in Figure 1, the relative amounts of FPR1-luciferase fusion proteins were very similar, with no statistical differences between the various haplotypes. Thus, the codon bias and codon pair bias differences based on the SNPs in the coding region of *FPR1* do not appear to affect the expression levels of the receptor in transfected U937 cells.

**Table 1:** Codon bias and codon pair bias scores for FPR1 variants calculated based on the codons for ten validated SNPs.

FPR1 haplotype	Amino acid number										Codon bias	Codon pair bias
	11	47	101	102	116	182	190	192	331	356		
1A	T	V	V	F-t	I-c	P-c	R	K	T-c	A	189.9	−0.900
1C	T	V	V	F-t	I-c	P-c	R	K	T-t	A	184.1	−1.107
2A	T	V	V	F-t	I-c	P-c	W	N-c	T-c	A	178.3	−1.854
3A	I	V	V	F-t	I-c	P-c	R	N-t	T-c	E	209.1	+0.443
3C	I	V	V	F-t	I-t	P-a	R	N-t	T-c	E	201.4	−0.783
4A	I	V	L	F-t	I-c	P-c	R	N-t	T-c	A	182.0	+1.085
5A	T	V	L	F-t	I-c	P-c	R	K	T-c	A	195.0	+0.471
6A	T	V	L	F-t	I-c	P-c	R	N-t	T-c	A	180.1	+0.932
8A	I	V	V	F-t	I-c	P-c	R	N-t	T-c	A	174.0	−1.765
9A	I	V	V	F-t	I-c	P-c	R	K	T-c	A	191.8	−0.747
10A	I	V	L	F-t	I-c	P-c	R	K	T-c	A	196.5	+0.625
11A	T	V	V	F-t	I-c	P-a	R	N-t	T-c	A	172.1	−1.918
12B	T	V	V	F-t	I-c	P-c	R	N-t	T-c	E	207.2	+0.380
12C	T	V	V	F-t	I-c	P-a	R	N-t	T-c	E	204.3	−1.098
12D	T	V	V	F-c	I-c	P-c	R	N-t	T-c	E	209.9	+2.346
16A	T	V	V	F-c	I-c	P-c	R	K	T-c	E	224.8	+1.796
25A	T	A	L	F-t	I-c	P-c	R	K	T-c	E	227.2	+1.202

Haplotype designations 1A-16A are by Sahagun-Ruiz *et al.* [2]. B, C and D show haplotypes in which the SNP does not change the amino acid compared to A [4]. The table includes the *FPR1* SNPs in the following order: *c.32C>T*/p.T11I, *c.140T>C*/p.V47A, *c.301G>C*/p.V101L, *c.306T>C*/p.F102F, *c.348C>T*/p.I116I, *c.546C>A*/p.P182P, *c.568A>T*/p.R190W, *c.576T>G>C*/p.N192K, *c.993C>T*/p.T331T, *c.1037C>A*/p.A356E. The codon bias results show the differences between the various haplotypes based on the total of each SNP codon usage score, as obtained from the GenBank *Homo sapiens* Codon Usage Database (http://www.kazusa.or.jp/codon/cgi-bin/showcodon.cgi?species=9606). The codon pair bias results show the differences between the various haplotypes based on the total of each SNP codon pair score, as calculated from the Supplemental Material by Coleman *et al.* www.sciencemag.org/cgi/content/full/320/5884/1784/DC1 [6]. Amino acids are shown in single letter code. The nucleotide in the 3^rd^ position of the synonymous codons is as shown.

### Genotyping of FPR1 promoter

The *FPR1* gene contains a single promoter region previously described by several groups [14], [18], [19]. The Human Genome Sequencing Project identified a single SNP in the *FPR1* promoter region, −56C>T (rs4802859), relative to the transcription start site (GenBank accession number NT_011109.16). To determine the relative frequency of this SNP, we carried out PCR amplification and sequencing of the promoter from 69 American Caucasians. We found that 18.8% of the individuals were homozygous for the −56T allele, similar to 15.9% in the European population reported by the HapMap project (http://www.ncbi.nlm.nih.gov/projects/SNP/snp_ref.cgi?rs=4802859). Our studies also revealed a second SNP in this region, −54G>C (rs62108945), with an occurrence of 5.8% heterozygous individuals and 0% homozygous individuals (Table 2). No genotyping data are available at this time in the GenBank for this SNP (http://www.ncbi.nlm.nih.gov/projects/SNP/snp_ref.cgi?rs=62108945). The most common genotype among Caucasians was −56C/C, −54G/G, with an occurrence of 45% (Table 2).

**Table 2:** SNP genotyping of the *FPR1* promoter in Caucasians.

−56 position	−54 position	Number of cases n = 69	Frequency
C/C	G/G	31	0.449
C/T	G/G	21	0.304
T/T	G/G	13	0.188
C/C	G/C	3	0.043
C/T	G/C	1	0.011
T/T	G/C	0	0.000
C/T	C/C	0	0.000
T/T	C/C	0	0.000

The SNP nucleotide positions are numbered based on the 5′-most transcription start site, as described by Murphy *et al.* [14].

### Localization of FPR1 promoter activity

**Figure 2:**
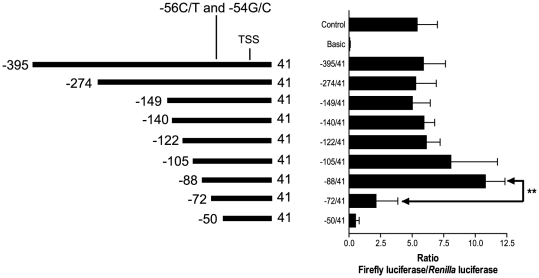
Identification of the minimal promoter region of *FPR1*. Serial deletion fragments of the *FPR1* promoter were generated by PCR and cloned upstream from the luciferase reporter gene in the pGL3 Basic vector. 10 µg of pGL3-Control vector containing the SV40 promoter was used as positive control and 30 µg of pGL3-Basic lacking a promoter was used to measure background luminescence. The amount of pGL3-Basic-*FPR1* promoter plasmids in all experiments was 30 µg. U937 cells were co-electroporated with the firefly luciferase plasmids and 300 ng of pRL-TK as a transfection standard. Results show the mean ratios of firefly to *Renilla* luciferase 24 hours post-transfection from 6–19 separate experiments ± S.E.M. Unpaired *t* test demonstrated that the luciferase activity of the −72/41 construct was significantly lower than the activity of the −88/41 construct, **p-value<0.01. Abbreviation: TSS, transcriptional start site.

To identify the minimal promoter region for transcriptional activity of *FPR1*, nine different *FPR1* promoter fragments ranging in size from 91 to 436 bp were cloned upstream of the luciferase reporter gene in vector pGL3 Basic (Figure 2). The nucleotide sequence was enumerated relative to the 5′-most transcriptional start site (TSS), designated nucleotide +1 [14]. The promoter constructs were co-transfected into U937 cells with the quantitative control vector, pRL-TK. Similar expression levels of firefly luciferase were observed with the five largest promoter fragments (−395/41, −274/41, −149/41, −140/41, and −122/41), whereas two smaller promoter fragments (−105/41 and −88/41) appeared to result in somewhat higher expression, although the differences were not statistically significant (Figure 2). Additional deletion of 16 nucleotides (−72/41), resulted in a significant drop in expression levels (Figure 2). Based on these results, the most important region for transcriptional activation of the *FPR1* gene appears to be between nucleotides −88 and −72.

### Mutagenesis studies of the FPR1 promoter

**Figure 3:**
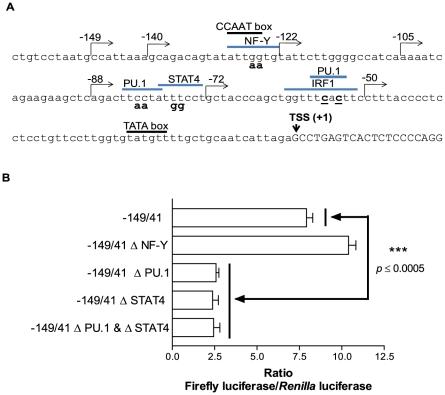
Location of putative transcription factor binding sites on *FPR1* promoter. **A.** Sequence analysis using PROMO3 software identified certain transcription factors commonly expressed in myeloid cells as putative regulators of *FPR1* transcription. The numbers indicate the first nucleotide of the various promoter constructs in relation to the transcriptional start site (TSS). The −56 and −54 SNPs are underlined and the various mutations of putative transcription factor binding sites are in bold. **B.** Site-directed mutagenesis of the putative PU.1 and STAT4 binding sites resulted in a significant decrease in firefly luciferase activity. U937 cells were co-transfected with the indicated wild-type and promoter mutant constructs and pRL-TK to normalize for transfection efficiency. Data show the mean ratios from three experiments ± S.E.M. One-way analysis of variance showed that differences in luciferase activity among the constructs were significant (*p* value<0.0001), and unpaired *t* test showed that the luciferase activities of each of the PU.1 and STAT4 mutant constructs were significantly lower than that of the wild-type construct, *p* value≤0.0005.

The *FPR1* promoter sequence between −149 and +1 was analyzed using the Promo3 software (http://alggen.lsi.upc.es/cgi-bin/promo_v3/promo/promoinit.cgi?dirDB=TF_8.3) to identify possible binding sites for transcription factors [20], [21]. We identified a putative NF-Y binding site at −129 to −123, a putative PU.1 binding site at −84 to −79, a putative STAT4 binding site at −79 to −73, a putative IRF1 binding site at −61 to −52 in the −56C/−54C promoter, and a putative PU.1 site at −59 to −53 in the −56C/−54C promoter (Figure 3). To examine whether the NF-Y, PU.1 and STAT4 sites are important for FPR1 expression, mutations in these sites were generated using the −149/41 C/G promoter construct. As shown in Figure 3B, the elimination of the putative NF-Y binding site (and CCAAT box) did not decrease the amount of firefly luciferase, but mutagenesis of the putative PU.1 and STAT4 binding sites, either individually or together, caused a significant decrease in transcriptional activity.

### In vivo and in vitro binding of PU.1 to FPR1 promoter

**Figure 4:**
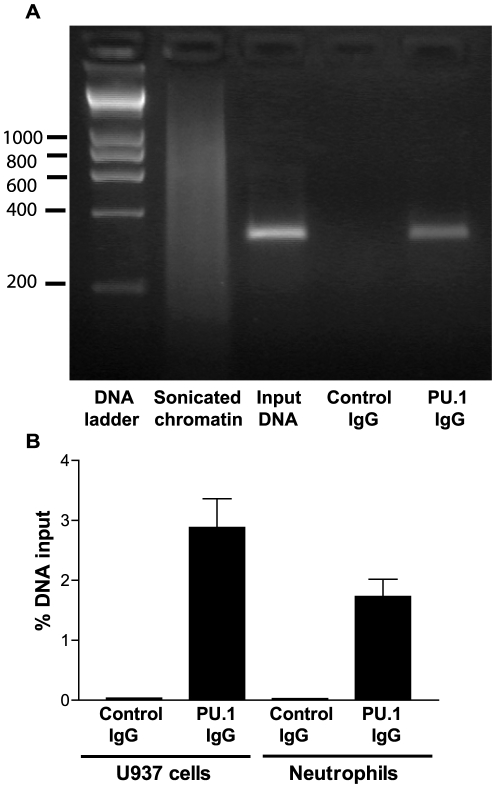
Confirmation of PU.1 binding to *FPR1* promoter by ChIP-qPCR. **A.** Cross-linked chromatin from U937 cells was sonicated to obtain an average DNA length of 600–800 bp. Immunoprecipitation was carried out using irrelevant control IgG or IgG against PU.1. The bands correspond to PCR products obtained amplifying a 324 bp fragment containing the putative PU.1 site (−87 to 237). The input DNA was obtained prior to the immunoprecipitation and represents ∼4% of the chromatin used in the immunoprecipitation. **B.** Cross-linked immunoprecipitated chromatin from U937 cells and human neutrophils was quantified by real-time qPCR and the amount of product was determined relative to the input chromatin. Each bar represents the mean ratio from three experiments ± S.E.M.

**Figure 5:**
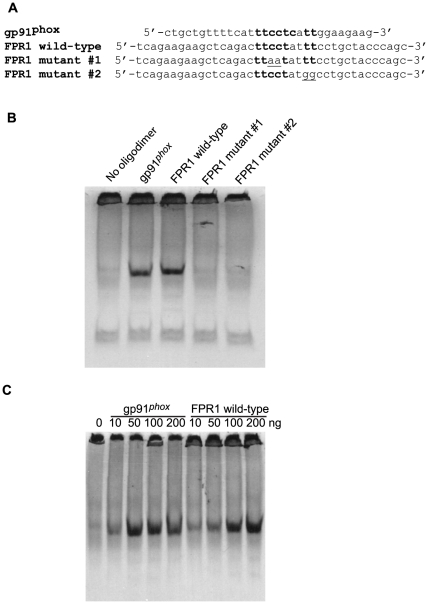
Confirmation of PU.1 binding to *FPR1* promoter by EMSA. **A.** The following oligonucleotide dimers were used in the binding assays: *gp91^pho^* ^x^ with a known PU.1 binding site (positive control); *FPR1* with a putative PU.1 binding site; two *FPR1* oligodimers with nucleotide substitutions (underlined) in the putative binding site. **B.** *In vitro* synthesized ^35^S-PU.1 was incubated with or without *gp91^phox^* and *FPR1* wild-type and mutant oligonucleotide dimers, as shown. **C.** Dose-dependence of ^35^S-PU.1binding was shown using 10–200 ng of *gp91^pho^* ^x^ and *FPR1* oligodimers.

Chromatin immunoprecipitation (ChIP) and quantitative real-time PCR (qPCR) were carried out to interrogate binding of PU.1 to the *FPR1* promoter. Chromatin fragments that bound to anti-PU.1 were PCR amplified with primers encompassing the −87 to +237 region of the promoter. As shown in Figure 4, U937 cells and human neutrophils showed a significant enrichment of the amplified *FPR1* promoter immunoprecipitated with the anti-PU.1 antibody compared to mock immunoprecipitation (Control IgG). Since our reporter assays suggested that the dual PU.1/STAT4 mutant did not result in further reduction in *FPR1* promoter activity compared to PU.1 or STAT4 alone, we examined the possibility that the second mutation in the putative STAT4 binding site may in fact inhibit the binding of PU.1. Our hypothesis gained further support upon examination of the nucleotide sequences immediately downstream of the known PU.1 binding sites of a number of promoters. As shown in Table 3, thymine is relatively conserved in positions 1, 2 and 3 immediately after the established PU.1 binding sequence, TTCCTC [16], [22]–[26]. To study the role of these thymines in the binding of PU.1 to the *FPR1* promoter, and to confirm the binding to the −84 to −76 site, we carried out electrophoretic mobility shift assays (EMSA) using wild-type and mutant promoter sequences, as shown in Figure 5A. Figure 5B shows ^35^S-PU.1 binding to an oligodimer containing the known *gp91^phox^* binding site (used as a positive control) [16] and to the *FPR1* wild-type oligodimer, but not to the mutant *FPR1* oligodimer containing two substitutions in the putative TTCCT PU.1 binding site (mutant #1). In addition, substitutions of two thymines with guanines downstream of the TTCCT site also prevented the binding of PU.1 (mutant #2). These results confirm the PU.1 binding in the *FPR*1 promoter and suggest that the binding region may include up to 9 nucleotides (TTCCTATTT). To compare the binding affinity of PU.1 to the *gp91^phox^* and the *FPR1* oligodimers, we carried out EMSA using various quantities of each oligodimer. As shown in Figure 5C, ^35^S-PU.1 appeared to bind to the gp91^phox^ oligodimer with somewhat higher affinity than to the *FPR1* oligodimer. The minor difference in binding affinity may in part be because position 6 of the *FPR1* binding site is adenine rather than cytosine, the more commonly observed nucleotide in this position (Table 3).

**Table 3:** PU.1 recognition in human neutrophil genes.

Gene	Sequence	Reference
MHC Class II	**TTCCTC**TTT	[22]
CD11b	**TTCCTC**TTT	[23]
gp40^phox^	**TTCCTC**TTA	[24]
gp47^phox^	**TTCCTC**TTT	[25]
gp67^phox^	**TTCCTC**TCT	[26]
gp91^phox^	**TTCCTC**ATT	[16]
FPR1	**TTCCT**ATTT	This study

The consensus sequence for PU.1 binding is shown in bold.

### Effect of −56/−54 SNPs on transcription factor binding

**Figure 6:**
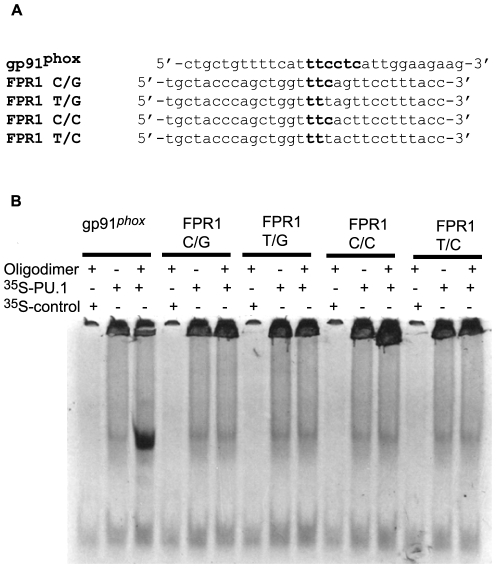
PU.1 does not bind the putative binding site in the −56/−54 SNP region of the promoter. **A.** Oligonucleotide dimers of *gp91^phox^* with a known PU.1 binding site and *FPR1* with the four possible −56/−54 SNP combinations were used in EMSA. **B.** *In vitro* synthesized ^35^S-PU.1 was incubated with *gp91^phox^* and the various *FPR1* oligonucleotide dimers. Where indicated, the incubation was carried out with a negative control (*in vitro* transcription/translation product using vector alone) or in the absence of oligodimer.

**Figure 7:**
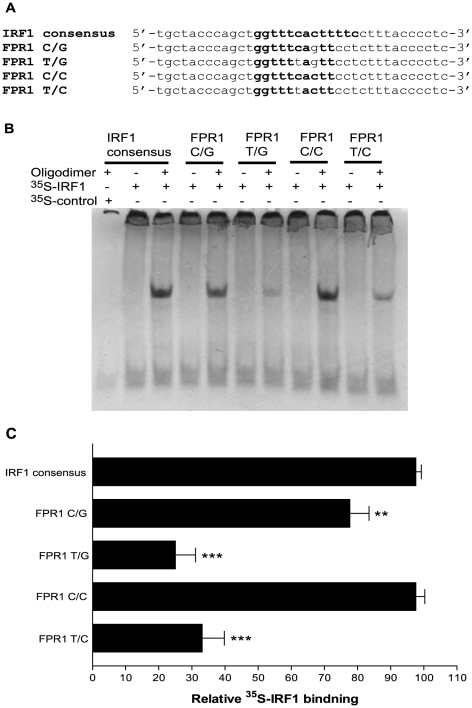
IRF1 binds the putative binding site in the −56/−54 SNP region of the promoter. **A.** Oligonucleotide dimers of IRF1 consensus binding sequence and *FPR1* with the four possible −56/−54 SNP combinations were used in EMSA. **B.** *In vitro* synthesized ^35^S-IRF1 was incubated with IRF1 consensus dimer and the various *FPR1* oligonucleotide dimers. Where indicated, the incubation was carried out with a negative control (*in vitro* transcription/translation product using vector alone) or in the absence of oligodimer. **C.** The binding of ^35^S-IRF1 to the various oligodimers was quantified by densitometry of the autoradiographic films. The results show the means ± S.E.M. from three experiments. One-way analysis of variance showed that the differences in luciferase activity among the *FPR1* constructs were significant (*P*<0.0001), and unpaired *t* test showed a significant difference between C/G and each of the other *FPR1* SNP constructs. **p-value<0.05, ***p-value<0.001.

**Figure 8:**
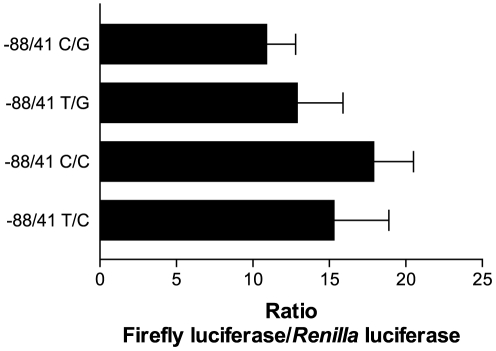
No significant differences in transcriptional activity between the four promoter variants were detected. *FPR1* minimal promoters −88/41 −56C/−54G, −56T/−54G, −56C/−54C and −56T/−54C were cloned upstream from the luciferase reporter gene, electroporated into U937 cells and expression was analyzed in a dual luciferase assay, as previously described. The graphs show the mean ratios from seven experiments ± S.E.M. The differences are statistically not significant in one-way analysis of variance, *p* value = 0.391.

As mentioned above, the PROMO3 analysis identified the −56/−54 SNP region of the *FPR1* promoter as another potential binding site for transcription factors. In particular, the −56C/−54C variant showed some homology with the consensus sequences for PU.1 and IRF1 binding (Figures 6A and 7A). Incubation with ^35^S-PU.1, however, did not result in binding to any of the *FPR1* −56/−54 variant oligodimers (Figure 6B). Thus, the *FPR1* minimal promoter region appears to contain only one PU.1 binding site located at nucleotides −84 to −76. EMSA using *in vitro* translated ^35^S-IRF1 resulted in good binding to the −56C/−54C oligodimer, slightly lower binding to the −56C/−54G oligodimer, and strongly reduced binding to −56T/−54G and −56T/−54C oligodimers (Figure 7B and 7C). To examine whether this difference in IRF1 binding to the *FPR1* promoter variants affects transcriptional regulation, we measured the promoter activity using the −88/41 *FPR1* minimal promoter construct with the various SNP combinations. The differences between the promoter variants were not statistically significant, suggesting that IRF1 does not play a major role in the transcriptional regulation of *FPR1* in U937 cells (Figure 8). This conclusion is also supported by the results with the −72/41 promoter showing significantly reduced reporter gene activity compared to the −88/41 promoter (Figure 2). The results were similar in the presence of interferon-γ, a inflammatory activator known to rapidly induce IRF1 expression in U937 cells [27], suggesting that the results shown in Figure 8 were not simply due to low levels of IRF1 in the cells (data not shown).

### Cell differentiation of U937 cells with DMSO and retinoic acid increase the promoter activity of FPR1

**Figure 9:**
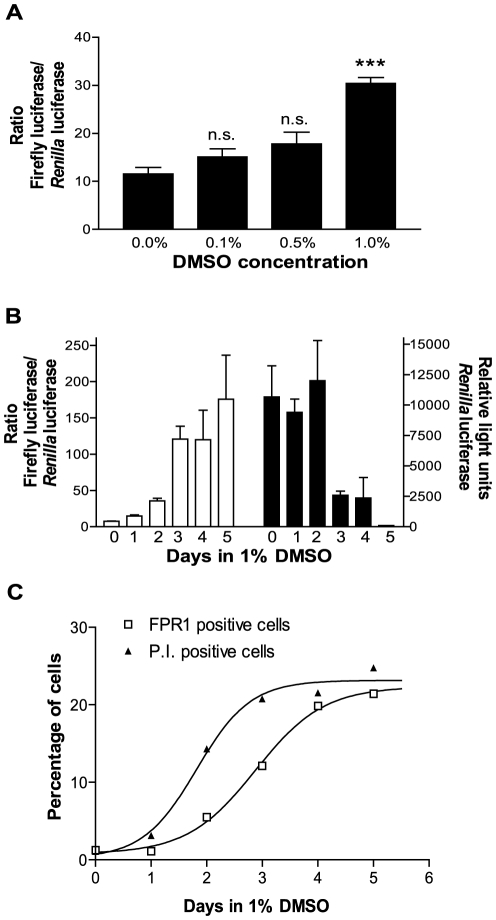
DMSO increases *FPR1* promoter-mediated transcription. **A.** Cells were co-transfected with pGL3 Basic-*FPR1* −149/41 and pRL-TK and incubated for 48 h in the presence or absence of DMSO prior to dual luciferase assay. The graphs show the mean ratios from four experiments ± S.E.M. Unpaired *t* test, *** *p*-value<0.0001. **B.** Cells were incubated for a total of 0–4 days in the presence of 1% DMSO prior to co-transfection with pGL3 Basic-*FPR1* −149/41 and pRL-TK. Cells were then incubated for another 24 h in the presence or absence of DMSO before dual luciferase assay. The white bars show the firefly luciferase/*Renilla* luciferase ratio, and the black bars show the *Renilla* luciferase activity in relative light units. The results show the mean ratios of triplicate samples ± S.E.M. **C.** U937 cells were incubated for 0–5 days in the presence of 1% DMSO prior to analysis by flow cytometry. Cells were incubated on ice for 1 h with 20 nM *N*-formyl-Nle-Leu-Phe-Nle-Tyr-Lys-fluorescein and 1 µg/ml propidium iodide, followed by analysis of 10,000 cells. The graph shows the percentage of cells that bound fluorescent ligand (FPR1 positive cells) and the percentage of dead cells (propidium iodide positive cells). The scatter plots can be seen in Figure S1 (Supporting Information).

**Figure 10:**
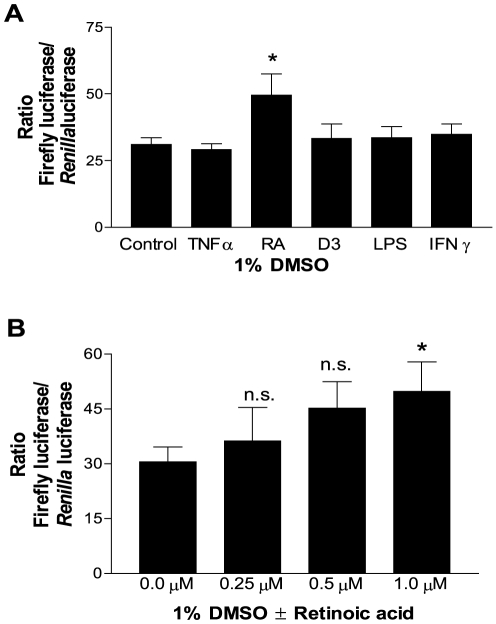
Retinoic acid in the presence of DMSO further increases *FPR1* promoter-mediated transcription. **A.** Cells were co-transfected with pGL3 Basic-*FPR1* −149/41 and pRL-TK and incubated for 48 h in the presence of 1% DMSO ± 100 U/ml tumor necrosis factor α (TNFα), 1 µM all-*trans* retinoic acid (RA), 10 nM 1,25 (OH)_2_-vitamin D3 (D3), 100 ng/ml lipopolysaccharide (LPS) and 500 U/ml interferon γ (IFNγ). The results are from three experiments ± S.E.M. Unpaired *t* test, * *p*-value<0.05. **B.** Cells were co-transfected with the pGL3 Basic-*FPR1* −149/41 plasmid and pRL-TK and incubated for 48 h in the presence of 1% DMSO ± various concentrations of all-*trans* retinoic acid (as shown). The graphs show the mean ratios from four experiments ± S.E.M. Unpaired *t* test, * *p*-value<0.05.

It has been previously established that U937 cells and HL-60 cells become differentiated in the presence of DMSO, resulting in expression of many immune receptors, including FPR1 [10], [11]. We confirmed the DMSO effect on FPR1 synthesis using our *FPR1* promoter −149/41 C/G-luciferase reporter construct (Figure 9A and 9B). A significant increase in activity could be detected 48 h after transfection in the presence of 1% DMSO compared to no DMSO (Figure 9A). The highest ratios were observed when cells were incubated in the presence of DMSO for 2–4 days prior to transfection (Figure 9B). The *Renilla* luciferase activity decreased with the longer incubation times in DMSO, however, with activity barely above background after 4 days in DMSO (Figure 9B), presumably because of a combination of increased cell death and lower electroporation efficiency [28]. Comparable results were obtained when the time-dependent effect of DMSO on endogenous FPR1 expression was examined in U937 cells by flow cytometry (Figure 9C and S1). We then examined the effect of various activating and priming agents on the transcriptional regulation of *FPR1* and found a statistically significant increase in the presence of all-*trans*-retinoic acid (RA), but not in the presence of tumor necrosis factor-α (TNFα), 1,25 (OH)_2_-vitamin D3 (D3), lipopolysaccharide (LPS), or interferon-γ (IFNγ) (Figure 10A). The effect of retinoic acid was concentration dependent, with a significant increase in reporter activity at 1 µM (Figure 10B).

## Discussion

Translation of eukaryotic proteins is regulated on multiple levels during initiation, elongation and termination. It is believed that the efficiency of translation elongation is in part determined by codon usage and the availability of tRNAs for infrequent codons. However, recent actively debated work in prokaryotes has shown that mRNA folding may play a predominant role in translation efficiency [29]–[31]. Similar studies in eukaryotes are few and controversial, but recent studies suggest that although codon bias plays an important role in translation efficiency [32], two-thirds of the variation in protein abundance can be attributed to mRNA abundance and sequence features related to translation and protein degradation [33]. The strongest individual correlates of protein expression were the length of the mRNA sequence, amino acid properties, upstream open reading frames and secondary structures in the 5′ untranslated region [33]. Studies of the cytoskeletal protein actin provided strong evidence for the importance of nucleotide content. The two isoforms of actin, β and γ, have almost indistinguishable amino acid sequences, but use alternate codons. γ-actin is translated more slowly than β-actin, exposing a lysine residue for ubiquitination, resulting in more rapid degradation of the protein [34]. Our studies focused on the hypothesis that codon bias and codon pair bias in *FPR1* gene variants may affect protein synthesis and/or stability. Our current results indicate that the codon differences and the variations in the amino acid properties of the different FPR1 haplotypes do not affect the abundance of FPRs. This confirms that translational regulation and protein stability are modulated by multiple factors and can be quite variable from one type of protein to another.

During myeloid hematopoesis, the *FPR1* gene becomes transcriptionally active. This study examined the transcriptional regulation of *FPR1*, with emphasis on characterizing the functional promoter, putative transcription factor binding sites, and the role of two SNPs in the promoter region. Traditionally, eukaryotic promoters contain different combinations of TATA boxes, CCAAT boxes, GC boxes, and other elements within 100–200 bp of the transcription initiation site [35]. No individual element is essential for promoter function, although one or more elements must be present for efficient initiation. Murphy and co-workers have previously identified a non-consensus TATA box (TATGTT), an inverted CCAAT box (ATTGG) and one pyrimidine-rich segment (−53 to −28) in this region of the *FPR1* promoter [14]. Our results suggest that the inverted CCAAT box is not utilized, since several promoter constructs lacking the site and also an inverted CCAAT box mutant (ATTAA) resulted in normal reporter gene activity. The strongest luciferase activity was obtained with a −88/41 promoter construct containing putative binding sites for PU.1 and STAT4 (−84 to −73). A deletion of 16 additional nucleotides (−72/41) resulted in 5-fold decrease in luciferase activity. Mutagenesis of the adjacent putative PU.1 and STAT4 binding sites either individually or together reduced reporter gene activity about 4-fold. ChIP-qPCR confirmed the *in vivo* binding of PU.1 to the *FPR1* promoter, and EMSA confirmed the PU.1 binding site. In addition, sequence comparisons with other known PU.1 binding promoter sequences and EMSA analysis showed that the PU.1 binding site may contain additional nucleotides, suggesting a consensus binding sequence of TTCCTCTTT (TTCCTATTT in *FPR1*). PU.1 is a member of the *ets* transcription factor family expressed in hematopoietic cells. It has been found at all stages of granulopoiesis, with the highest levels in PMNs [36], [37], and plays an important role in innate immune functions of these cells. Perhaps the most notable example is the multicomponent NADPH oxidase system. PU.1 has been found to be an essential activator for the expression of several of its components, including p47^phox^, gp91^phox^, p67^phox^, and p40^phox^ [24]–[26], [38].

The discovery of two SNPs at a distance of 54 and 56 nucleotides upstream from the transcriptional start site of *FPR1* suggested that they may be involved in transcriptional regulation of the gene. Many promoter SNPs have indeed been shown to affect protein expression, resulting in major health-related effects. For example, an SNP in the matrix metalloproteinase-12 (MMP-12) promoter influences the binding of transcription factor AP-1 and is associated with coronary artery disease [39], and a SNP in the promoter region of interleukin 4 (IL4) affects the binding of transcription factor NFAT, resulting in a 3-fold difference in IL4 expression [40]. We therefore examined the possibility that the −56/−54 SNPs affect transcription factor binding and protein expression. To do this, we compared the luciferase activity of the −88/41 promoter construct containing all four possible SNP combinations. The differences between the various constructs were not statistically significant, suggesting that this region is not critical for transcription. This conclusion was further supported by the finding that the −88/41 (C/G) and −72/41 (C/G) promoters showed a 5-fold difference in luciferase activity, indicating that the major regulatory domain is between nucleotides −88 and −72. However, we cannot completely rule out the possibility that the SNP region may under certain conditions contribute to the transcriptional regulation of *FPR1*, since *in vitro* studies suggested that IRF1 transcription factor preferentially binds the −56C/−54C sequence. Several potential IRF1 host defense target genes have been previously characterized, including the NADPH component, gp91^phox^ [16], [41].

Previous studies examining the transcriptional regulation of cell surface proteins involved in the differentiation and inflammatory response of myeloid cells, such as the various components of NADPH oxidase, have identified a number of cytokines, differentiation factors and bacterial components that up- or down-regulate expression. The most commonly studied are TNFα, retinoic acid, 1,25 (OH)_2_-vitamin D_3_, LPS and IFNγ. For example, the gene expression of the phagocyte cytosolic protein *p47^phox^* component of NADPH oxidase is up-regulated by TNFα, retinoic acid, 1,25 (OH)_2_-vitamin D_3_, and LPS, but down-regulated by IFNγ [42]–[44]. In contrast, IFNγ induces the expression of FcR and certain chemokine receptors (CCR1, CCR3 and CCR5) in U937 cells [45], [46]. Our results confirmed the previous results showing maturation and increased FPR1 expression in myeloid cells upon incubation with DMSO [10], [11]. Incubation with inflammatory activators in the absence of DMSO did not result in significant increases in *FPR1* promoter activity, and only retinoic acid, but not TNFα, 1,25 (OH)_2_-vitamin D_3_, LPS or IFNγ, further increased protein expression in the presence of DMSO. Thus, unlike some of the other innate immune receptors and molecules, the expression of FPR1 is dependent on cell differentiation and maturation rather than an inflammatory stimulus. Physiologically, this appears logical since FPR1 is one of the first receptors that is alerted to the presence of microorganisms, and directs the PMNs to the site of infection through chemotaxis. A cell equipped to immediately respond to a bacterial threat likely minimizes the damage by the invading microorganisms.

## Supporting Information

Figure S1 **Analysis of FPR1 expression in U937 cells by flow cytometry.** U937 cells were incubated 0–5 days with 1% DMSO, as indicated in the figure. FPR1 expression was visualized using a fluorescent high affinity binding ligand, *N*-formyl-Nle-Leu-Phe-Nle-Tyr-Lys-fluorescein (FL-1). Propidium iodide was used to visualize the non-viable cells (FL-2). The proportion of both FPR1-positive cells and non-viable cells increased over time with maximal FPR1 expression after 4–5 day incubation.
(EPS)
